# Antifungal and Antiaflatoxigenic Activities of Massoia Essential Oil and C10 Massoia Lactone against Aflatoxin-Producing *Aspergillus flavus*

**DOI:** 10.3390/toxins15090571

**Published:** 2023-09-16

**Authors:** Yubin Lee, Soo Jean Park, Kyeongnam Kim, Tae-Oh Kim, Sung-Eun Lee

**Affiliations:** 1 Department of Integrative Biology, Kyungpook National University, Daegu 41566, Republic of Korea; fnrl456@knu.ac.kr; 2 Applied BioSciences, Macquarie University, North Ryde, NSW 2109, Australia; soojean.park@mq.edu.au; 3 Institute of Quality and Safety Evaluation of Agricultural Products, Kyungpook National University, Daegu 41566, Republic of Korea; kn1188@knu.ac.kr; 4 Department of Environmental Engineering, Kumoh National Institute of Technology, Gumi 39177, Republic of Korea; 5 Department of Applied Biosciences, Kyungpook National University, Daegu 41566, Republic of Korea

**Keywords:** massoia essential oil, C10 massoia lactone, *Aspergillus flavus*, aflatoxin B_1_

## Abstract

Fungal infection and mycotoxin contamination are major hazards to the safe storage and distribution of foods and feeds consumed by humans and livestock. This study investigated the antifungal and antiaflatoxigenic activities of massoia essential oil (MEO) and its major constituent, C10 massoia lactone (C10), against aflatoxin B (AFB)-producing *Aspergillus flavus* ATCC 22546. Their antifungal activities were evaluated using a disc diffusion assay, agar dilution method, and a mycelial growth inhibition assay with the AFB analysis using liquid chromatography triple quadrupole mass spectrometry. MEO and C10 exhibited similar antifungal and antiaflatoxigenic activities against *A. flavus*. C10 was a primary constituent in MEO and represented up to 45.1% of total peak areas analyzed by gas chromatography–mass spectrometry, indicating that C10 is a major compound contributing to the antifungal and antiaflatoxigenic activities of MEO. Interestingly, these two materials increased AFB production in *A. flavus* by upregulating the expression of most genes related to AFB biosynthesis by 3- to 60-fold. Overall, MEO and C10 could be suitable candidates as natural preservatives to control fungal infection and mycotoxin contamination in foods and feeds as Generally Recognized As Safe (GRAS) in the Flavor and Extract Manufacturers Association of the United States (FEMA), and MEO is a more suitable substance than C10 because of its wider range of uses and higher allowed concentration than C10.

## 1. Introduction

Aflatoxins are one of the primary mycotoxins produced mainly by the genus *Aspergillus*, which exert liver cancer-causing effects in humans [1,2]. Aflatoxins consist of four major analogs, viz., aflatoxin B_1_ (AFB_1_), aflatoxin B_2_ (AFB_2_), aflatoxin G_1_ (AFG_1_), and aflatoxin G_2_ (AFG_2_). The biosynthetic pathways of these analogs have been well documented. *Aspergillus flavus* produces only AFB_1_ and AFB_2_, while *A. parasiticus* is capable of generating all four analogs [3,4,5]. Among their metabolites, AFB_1_, when consumed by livestock, can be degraded into AFM_1_; moreover, the degradation of AFB_1_ to AFM_1_ has been reported in human breast milk, which can then be transferred from mothers to their infants [6,7]. Therefore, several countries, including Canada, the EU, Korea, and the USA, have implemented strict regulations and set the maximum residue levels for aflatoxins in agricultural and dairy products [8,9]. Humans and livestock are easily exposed to these mycotoxins through the consumption of AFB-contaminated agricultural and dairy products and feedstocks during the preharvest or postharvest process [10,11,12]. Hazard analysis and critical control points (HACCP) have been introduced to eliminate the contamination of mycotoxins, including aflatoxins, and reduce their risk [13,14]. However, contamination with mycotoxins may occur based on ethnic practices related to the preparation and consumption of agricultural and dairy products [15,16]. For example, in Korean doenjang, which is a fermented soybean paste that is traditionally dried under sunlight for a long winter time, a higher amount of aflatoxin contamination can be found when it is produced using natural strains for inoculation [15,16]. Aflatoxin, among mycotoxins, has been frequently reported to exceed the permissible levels worldwide in numerous cases, and various methods are also employed to prevent contamination with aflatoxin and its precursors [17,18,19]. 

Control methods for aflatoxin include the use of a fungicide to directly sterilize mycotoxin-producing fungal species, the removal and detoxification of aflatoxins from contaminated agricultural products and feeds, and the biological control of aflatoxin-producing *Aspergillus* sp. in agricultural products [4,20,21]. Naturally occurring products such as monoterpenes and organic acids have recently been used to reduce or minimize aflatoxin contamination in foods and feeds [22,23,24,25]. These substances can serve as food preservatives [24,26]. 

Massoia essential oil (MEO, *Massoia aromatica* Becc, Lauraceae) has shown an antifungal effect on immunosuppression-related infection of *Candida albicans* at an IC_50_ value of 0.074% (*v*/*v*) [27]. Its toxic action is primarily associated with the suppression of biofilms rather than the inhibition of hyphal growth in *C. albicans*. Similarly, the main component of MEO, C10 massoia lactone (C10), showed no inhibitory effect on hyphal development at the tested concentrations [27]. Yuan et al. [28] developed massoia lactone-loaded and food-grade nanoemulsions and evaluated their antifungal activity against *Metschnikowia bicuspidate*, a pathogenic yeast that causes milky disease in the Chinese mitten crab. Furthermore, apart from demonstrating antifungal activities against pathogenic yeasts, both MEO and C10 also possess anticancer and anti-inflammatory effects [29,30]. Recently, Zhang et al. [31] demonstrated the inhibitory effect of C10 on the growth of 21 crop pathogens, including *A. flavus*. In the study, the mode of toxicity was attributed to a reduction in ergosterol content and an increase in reactive oxygen species content, leading to cellular necrosis and cell death. However, the study did not evaluate how aflatoxin production affects the measurement of aflatoxin concentration within the tested range of C10. 

In this study, the antifungal activities of MEO and C10 against *A. flavus* ATCC 22546 grown in solid and liquid growth mediums were evaluated to determine the growth inhibition rates. Additionally, MEO was analyzed using GC–MS to confirm the primary components, and a cross-check determination was conducted using the Kovats retention index with commercially available references. Aflatoxin production under chemical treatments was analyzed by liquid chromatography in conjunction with a fluorescent detector or an MS/MS detector. *A. flavus* produced AFB_1_ and AFB_2_ but not AFG_1_ and AFG_2_. Therefore, the antiaflatoxigenic activities of MEO and C10 in this study were attributed to the inhibition of AFB_1_ and AFB_2_ production. Furthermore, the expression of aflatoxin biosynthesis-related genes was analyzed to gain insights into the role of MEO and C10 in inhibiting aflatoxin biosynthesis. 

## 2. Results and Discussions

### 2.1. Antifungal Activities of MEO and C10 against A. flavus

**Figure 1:**
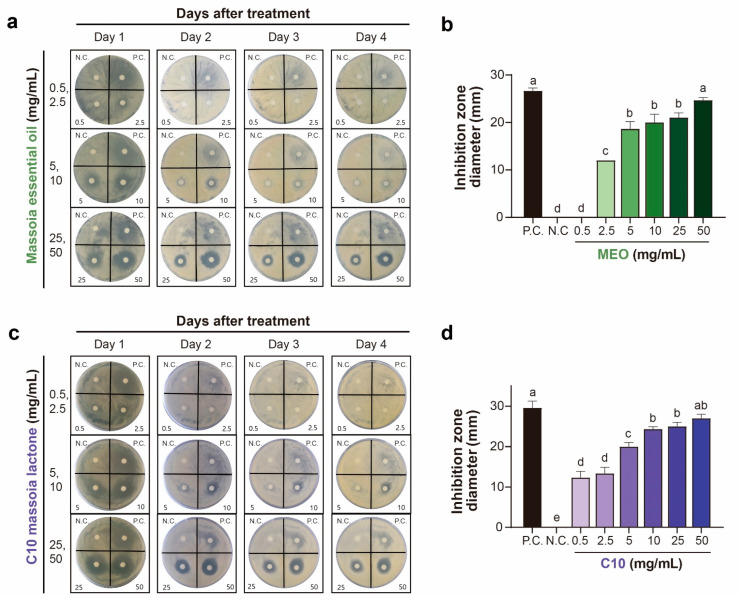
Antifungal activities of massoia essential oil (MEO) and C10 massoia lactone (C10) using the disc diffusion assay at the treated concentrations in the range of 0.5 to 50 mg/mL on *Aspergillus flavus* ATCC 22546 during 4 day incubation. (**a**,**c**) Picture of antifungal activities of MEO or C10 over the incubation time (day). (**b**,**d**) Inhibition zones of fungal growth at 1 day incubation with statistical analysis. N.C., negative control (DMSO); P.C., positive control (azoxystrobin 1 mg/mL). All experiments were performed in triplicates. Statistical analyses were performed using one-way ANOVA, followed by post-hoc Tukey’s test. Different lowercase letters indicate significant differences among groups: a > b > c > d > e.

The antifungal activities of MEO and C10 were determined using three different antifungal assay methods, viz., the disc diffusion assay, the agar dilution method, and the mycelial growth assay. In the disc diffusion assay, MEO exhibited strong antifungal activities against *A. flavus* in the tested concentration range of 2.5–50 mg/mL (Figure 1 and Figure S1). 

At 2.5 mg/mL, MEO exhibited significant antifungal activity, reaching approximately half of that exhibited by the positive control azoxystrobin (1 mg/mL), which is the currently used fungicide. In contrast, C10 exhibited its antifungal activity at a lower concentration of 0.5 mg/mL (Figure 1 and Table S1). Interestingly, the antifungal activities of MEO and C10 were sustained for up to 4 days after treatment, whereas the positive control lost its antifungal activity after 2 days, as indicated by the disappearance of the inhibitory zone (Figure 1). This indicates that antifungal activities of MEO and C10 to *A. flavus* ATCC 22546 can have a prolonged effect. Higher concentrations of MEO and C10 exhibited similar effects on the growth of *A. flavus* in the disc diffusion assay (Figure 1 and Figure S1). 

**Figure 2:**
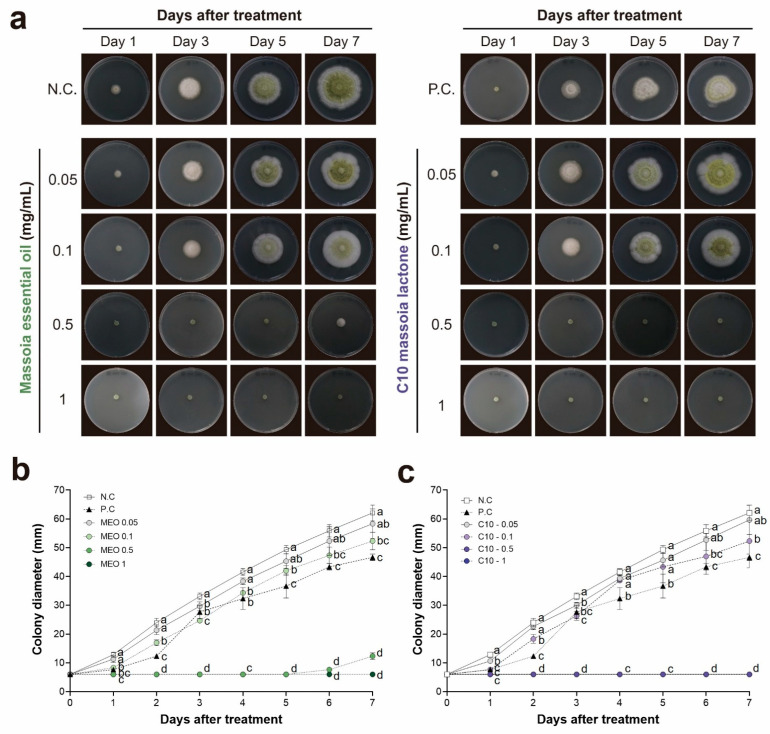
Antifungal activities of massoia essential oil (MEO) and C10 massoia lactone (C10) using agar dilution method at the treated concentrations in the range of 0.05 to 1 mg/mL on *Aspergillus flavus* ATCC 22546 during 7 days-incubation. (**a**) Picture of antifungal activities of MEO or C10 over the incubation time (day). N.C., negative control (DMSO); P.C., positive control (azoxystrobin 0.1 mg/mL). (**b**,**c**) Colony diameter for 7 days after treatment. All experiments were performed in triplicates. Statistical analyses were performed using one-way ANOVA, followed by post-hoc Tukey’s test. Different lowercase letters indicate significant differences among groups: a > b > c > d.

In the agar dilution method, MEO demonstrated potent antifungal effects against *A. flavus* within the concentration range of 0.1–1 mg/mL (Figure 2 and Table S2). Meanwhile, C10 displayed marginally superior antifungal activity compared to MEO at an identical concentration of 0.5 mg/mL. The growth of *A. flavus* was completely inhibited over 1 mg/mL of MEO and C10 until 7 days after treatment (Figure 2). Notably, the effective concentration range identified via the agar dilution method was over ten times lower than that observed in the disc diffusion assay (Figure 1 and Figure 2). These findings suggested that MEO and C10 play a role in inhibiting *A. flavus* as volatile compounds.

**Figure 3:**
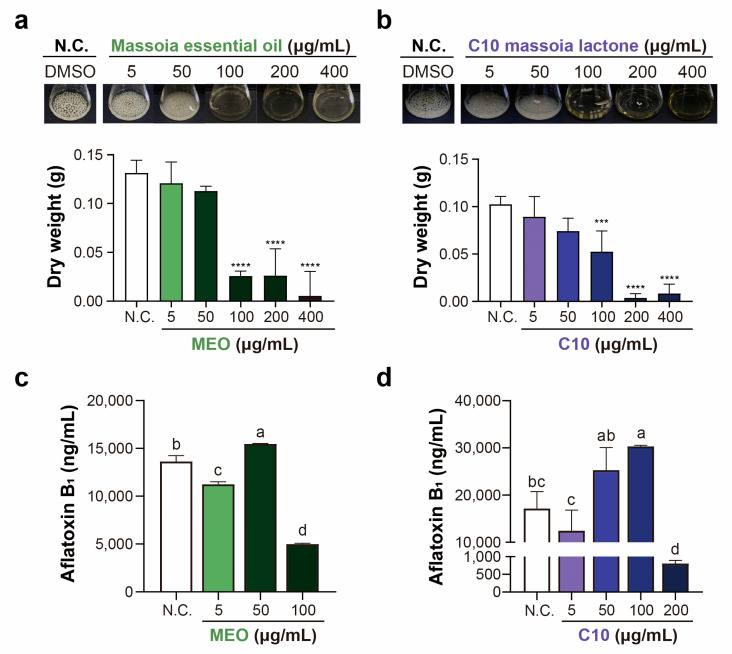
Antifungal properties of massoia essential oil (MEO) (**a**) and C10 massoia lactone (C10) (**b**) toward *Aspergillus flavus* ATCC 22546 grown in a liquid medium. DMSO was used as a solvent control for this study. *A. flavus* ATCC 22546 produced aflatoxin B_1_, and its measurements using LC-MS/MS were performed. Inhibitory effects on aflatoxin B_1_ production were found by MEO (**c**) and C10 (**d**). All experiments were performed in triplicates. Statistical analyses were performed using one-way ANOVA, followed by post-hoc Tukey’s test. Asterisks in drying weight data indicate significant differences from the control. Different lowercase letters indicate significant differences among groups: a > b> c> d.

In the mycelial growth assay, the antifungal activities were determined in the concentration range of 5–400 μg/mL of MEO and C10 (Figure 3). At 100 μg/mL, both MEO and C10 exhibited strong and similar levels of antifungal activities on *A. flavus* mycelial growth. At both 200 and 400 μg/ mL, C10 exhibited stronger antifungal activity than MEO.

Recently, there has been growing attention to natural products as replacements for chemical preservatives. Among these, certain natural products, especially plant essential oils, demonstrated strong antifungal activities, often requiring lower concentrations when incorporated into foods [32]. Similarly, this study evaluated the antifungal activity of MEO on *A. flavus* growth. The strong antifungal activity of MEO was confirmed using three bioassays, viz., disc diffusion bioassay, agar dilution method, and mycelial growth assay (Figure 1, Figure 2 and Figure 3). Within these three assays, MEO exhibited its antifungal activity from 2.5 mg/mL in the disc diffusion bioassay and 0.1 mg/mL in both the agar dilution method and mycelial growth assay. The used concentrations of 2.5 and 0.1 mg/mL are equivalent to 0.25% and 0.01%, respectively. When these values were compared to those of organic acids, the antifungal activity observed in the mycelial growth assay was found to be 5- and 50-fold stronger than those of benzoic acid and propionic acid, respectively [26]. This result is consistent with that of C10, a major component in MEO (Table 1). A previous study showed that C10 exhibited strong antifungal activity against crop and food pathogens, including *A. flavus* [31]. 

Chemical control methods have been employed to combat fungal infections and eradicate fungal species responsible for plant and animal diseases and the contamination of stored or distributed foods. Synthetic fungicides are widely used in crop fields to control phytopathogenic fungi, proving to be effective in crop cultivation until harvest [33]. However, the development of resistance in the target fungi poses a significant challenge to the continued use of fungicides in the future. For instance, sterol 14α-demethylase inhibitors inhibit fungal ergosterol biosynthesis. These inhibitors typically contain triazoles consisting of a five-membered di-unsaturated ring moiety with three nitrogen atoms. Several fungal species have demonstrated the ability to develop resistance to these triazole compounds [34]. 

In contrast to phytopathogenic fungicides, a different set of fungicides such as polyenes (for example, amphotericin B), echinocandins, and azoles is used to control the three most lethal human fungal pathogens, including *Candida* spp., *Cryptococcus* spp., and *Aspergillus* spp. [35]. A similar obstacle exists to prolonging the use of these three types of fungicides on human fungal pathogens due to the development of fungicidal resistance [36]. Contamination with fungal mycotoxins, such as AFB_1_, ochratoxin A, deoxynivalenol, zearalenone, and fumonisin B_1_, exacerbates fungal infections in food [37]. Strategies for controlling fungal infections in foods have evolved to address environmental concerns by chemical fungicides, to adapt to stricter regulations on fungicide use, and to respond to market trends that demand new food-preservation agents [38]. Davies et al. [38] suggested biocontrol and natural products as alternative methods to control fungal infection and introduced “clean label” food products with the use of natural product preservatives.

Various preservatives are used to control fungal infections on foods and feeds, including propionic acid, sorbic acid, and benzoic acid [39]. The antifungal and antiaflatoxigenic activities of these organic acids have been well investigated, and their ability to reduce aflatoxin production via suppressing aflatoxin-producing genes has been elucidated [26]. However, achieving effective control of *A. flavus* necessitates the use of high treatment concentrations, such as 0.05% benzoic acid and 0.1% sorbic acid, and even propionic acid at a concentration of 0.5% into the mycelial growth medium to achieve 100% control [26]. 

### 2.2. GC–MS Analysis of MEO Constituents Using RI

**Figure 4:**
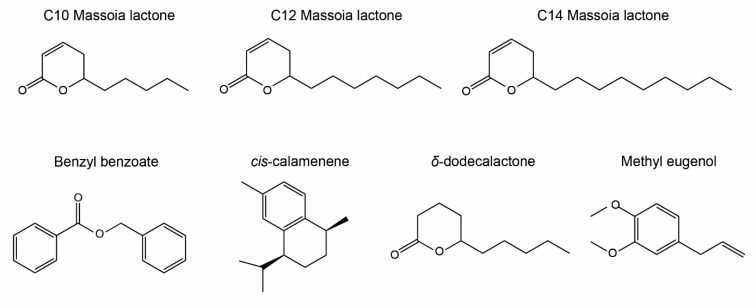
Chemical structures of the main components in massoia essential oil.

GC–MS analysis of MEO constituents was conducted to verify active compounds quantitatively and qualitatively using two different analytical columns, SH-Rtx-5MS and FAMEWAX, under the same GC running conditions. C10 was detected at the highest concentration in MEO, reaching up to 45.2% of total peak areas, followed by C12 massoia lactone (C12), reaching up to 36.7% of total peak areas (Table 1 and Figure 4). Another type of lactone, C14 massoia lactone (C14), was also detected in 1.4% of the MEO sample (Table 1). However, C10 is only a commercially available reference, and C12 and C14 are not available in the market; thus, their reference retention index (RI) was not provided. Benzyl benzoate was detected as the third abundant compound (3.8%), followed by cis-calamenene (3.2%) and δ-dodecalactone (1.9%). Methyl eugenol was detected in minute quantity (0.3%), which possesses antifungal activity due to its phenylpropanoid moiety (Table 1 and Figure 4).

### 2.3. Aflatoxin Production after MEO and C10 Treatments in A. flavus

AFB_1_ was analyzed in *A. flavus* mycelial growth medium after treatment with both MEO and C10. Notably, the concentrations of AFB_1_ were significantly decreased at 100 μg/mL of MEO (Figure 3c). This result is consistent with the results of mycelial growth inhibitory assays, in which MEO significantly inhibited *A. flavus* growth at 100 μg/mL concentration. Regarding the treatment with C10, a significant reduction in the AFB_1_ concentration was observed at a concentration of 200 μg/mL (Figure 3d), which aligns with the notable inhibition of *A. flavus* mycelial growth at the same concentration. Consequently, the addition of both MEO and C10 to the *A. flavus* growth medium led to a significant suppression of aflatoxin production. 

The antiaflatoxigenic activities of MEO and C10 against AFB-producing *A. flavus* ATCC 22546 became evident at a concentration of 0.1 mg/mL for both materials (Figure 2 and Figure 3). This finding is particularly intriguing as it indicates that chemical treatment can suppress AFB production even before the complete inhibition of growth in the mycelial growth assay. For instance, Moon et al. [26] demonstrated that propionic acid suppressed 99% of AFB production at 0.1% treated concentration, where it inhibited approximately 50% of mycelial growth under the same experimental conditions. This result extends to treatments with other acids, such as acetic acid, benzoic acid, and butyric acid [26]. Plumbagin, a representative naphthoquinone, demonstrated approximately 20% inhibitory effect on the mycelial growth of *A. flavus* ATCC 22546 at 10 mg/L, while the AFB_1_ production decreased to 30% compared to that in the control group [25]. 

### 2.4. Differential Expression of Aflatoxin-Producing Genes after Chemical Treatments

The expression levels of AFB biosynthesis-related genes were assessed through qRT-PCR in response to treatment with both materials. For MEO treatment, samples were collected from the mycelial growth medium up to a concentration of 50 μg/mL of MEO due to limited fungal sources for qRT-PCR analysis at higher concentrations. On the other hand, for C10 treatment, the samples were collected up to a treatment concentration of 100 μg/mL, as sufficient mycelial growth was available for analysis. 

**Figure 5:**
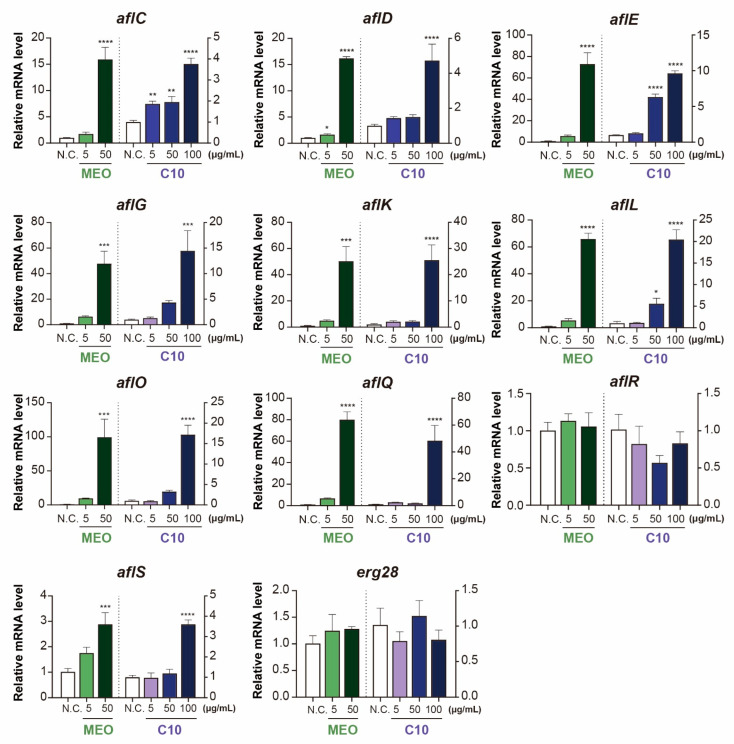
Gene expression levels after massoia essential oil (MEO) and C10 massoia lactone (C10) treatments toward *Aspergillus flavus* ATCC 22546 grown in a PDB liquid medium. All experiments were conducted three times. *β-tubulin* was utilized for the normalization of genes, and gene responsibilities were described in Table S3. All data are expressed as mean ± standard deviation (SD). * *p* <  0.05; ** *p* < 0.01; *** *p* < 0.001; **** *p* < 0.0001.

The qRT-PCR analysis revealed that only two genes, viz., *aflR* and *erg28*, remained unchanged after the treatments, which encoded an AFB-producing transcription regulator and 14α-demethylase, respectively (Figure 5). The most striking outcome was the upregulation observed in *aflE*, *aflG*, *aflK*, *aflL*, *aflO*, and *aflQ* at 50 μg/mL of MEO and 100 μg/mL of C10. Their altered expression ranged from a 40 to 100-fold increase compared to that in the control group. Despite the significant changes in gene expression, the AFB production in absolute mycelial amount was too low, as shown in Figure 3. These findings demonstrate that *A. flavus* responds to MEO and C10 with upregulation of AFB biosynthesis pathways. Furthermore, other genes such as *aflC*, *aflD*, and *aflS* were also upregulated by a factor of 3 to 15-fold after treatments with both materials (Figure 5). 

Interestingly, some AFB biosynthesis-related genes exhibit distinct responses to chemical stress. For instance, after 5 μg/mL of plumbagin treatment in *A. flavus* mycelial medium, *aflG*, *aflK*, *aflL*, and *aflQ* were upregulated more than two-fold compared with the control, whereas AFB_1_ and AFB_2_ production reached only 30% of the control group [25]. These findings show that AFB production was not affected by the upregulation of some genes related to AFB biosynthesis in *A. flavus*. However, upon treatment with 25 mg/L of other naphthoquinones, such as vitamin K_3_ (menadione), all tested genes associated with AFB biosynthesis were upregulated in the range of 2 to approximately 60-fold, leading to a higher production of AFB_1_ (244%) and AFB_2_ (269%) [25]. This result aligns closely with our results, showing that MEO and C10 upregulated all genes except *aflR* in a similar manner (Figure 5). The two compounds vitamin K_3_ and C10 similarly function on the AFB biosynthesis pathway in *A. flavus*, indicating that *A. flavus* has evolved in a similar manner in response to these chemical stresses. 

MEO has been considered by the Flavor and Extract Manufacturers Association of the United States (FEMA) as a Generally Recognized As Safe (GRAS) substance, and it can be used as a food additive for baked products (50 ppm), soft candy (30 ppm), and nonalcoholic/alcoholic beverages (20 ppm) [40]. Therefore, its use as a natural preservative in the food industry is encouraged to prevent fungal infection and mycotoxin contamination, especially *A. flavus* infection and AFB contamination. C10 is also a FEMA GRAS substance that can be used in baked products (1.0 ppm), soft candy (0.5 ppm), and nonalcoholic beverages (0.5 ppm) [40]. However, the antifungal and antiaflatoxigenic activities of C10 were equivalent or lower to those of MEO; therefore, MEO would be a preferred choice as a natural preservative for controlling fungal infections and preventing mycotoxin contamination in foods. In addition to this regard, manufacturing costs would be more expensive as it needs extensive purification from MEO or massoia bark resources. 

Environmental issues associated with the use of current food additives are capturing large public attention. For instance, a study revealed the presence of 13 food additives in 10 tested swimming pools, with the most commonly identified additives being antioxidants, including E320 (butylated hydroxyanisole) and E321 (butylated hydroxytoluene), and preservatives, including E211 (sodium benzoate) and E210 (benzoic acid) [41]. This shows that a vast range of contamination of food additives has occurred in public spaces. Furthermore, plumbagin as a future medicinal candidate for treating cancers resulted in approximately 50% mortality at a tested concentration of 0.625 mg/L with significant malformations such as curved spines and developmental delays in zebrafish [25]. Therefore, a thorough evaluation of the environmental toxicity of MEO and C10 is an essential prerequisite before their use as preservatives in the food industry. 

## 3. Conclusions

The potent antifungal capabilities of MEO against AFB-producing *A. flavus* ATCC 22546 have been affirmed through three distinct bioassays: disc diffusion assay, agar dilution method, and mycelial growth assays. In these evaluations, MEO exhibited its antifungal activity at concentrations of 2.5 mg/mL in the disc diffusion assay and 0.1 mg/mL in both the agar dilution method and mycelial growth assays. GC-MS analysis revealed C10 massoia lactone (C10) as a primary component of MEO, suggesting its pivotal role in MEO’s antifungal and antiaflatoxigenic attributes. C10 demonstrated comparable antifungal and antiaflatoxigenic actions against *A. flavus*, mirroring the concentrations observed for MEO. Our results emphasize the remarkable antifungal and antiaflatoxigenic activity of both MEO and C10 against *A. flavus* ATCC 22546. Their potential to serve as powerful natural preservatives may mark a significant shift in addressing fungal contamination in the food sector. When applied practically, MEO and C10 can be incorporated into baked products at concentrations of 50 mg/L and 5 mg/L, respectively, and are recognized as GRAS substances. Although both compounds show promise, MEO seems to be the preferable option for direct incorporation into food. 

## 4. Materials and Methods

### 4.1. Chemicals 

MEO was purchased from Escentials of Australia (Noosaville, Queensland, Australia). 6-Pentyl-5,6-dihydropyran-2-one (known as C10 massoia lactone, ≥95%) was obtained from Sigma-Aldrich Co. (St. Louis, MO, USA). Furfural, linalool, methyl eugenol, benzyl benzoate, and benzyl salicylate were acquired from Sigma-Aldrich Co. (St. Louis, MO, USA). 

### 4.2. Preparation of Fungal Cultures 

*A. flavus* ATCC 22546 was purchased from the KCCM (Korea Culture Center of Microorganisms, Seoul, Republic of Korea). A subculture for the fungi and disc diffusion assay was undertaken in potato dextrose agar (PDA, Difco, Franklin Lake, NJ, USA). For the liquid culture of the fungi, potato dextrose broth (PDB, Difco, Franklin Lake, NJ, USA) was utilized, and the incubation of *A. flavus* was conducted at 25 °C ± 2 °C for 5 days on PDA medium. A spore suspension (10^7^ spores/mL) was prepared by slowly shaking the plate with the addition of Tween 80 solution (0.1%).

### 4.3. Antifungal Disc Diffusion Assay 

Antifungal activities of MEO and C10 were undertaken in Petri dishes on a PDA medium, followed by a previous study [25]. Briefly, a suspension of the fungi spore (10^7^ spores/mL) was spread on the PDA media, and 4 paper discs (6 mm) were put on the surface layer of the agar plate. Various concentrations (ranges between 0.5 mg/mL and 50 mg/mL) of MEO and C10 were spiked on paper discs and left for 4 days at 25 ± 2 °C. The inhibitory circle zones were measured, and the antifungal activities of MEO and C10 were compared with the currently used fungicide, azoxystrobin, as positive controls at a concentration of 1 mg/mL. Negative controls were treated using DMSO, which is the same solvent employed to dilute MEO and C10. Each treatment was conducted with three replicates and treated with 15 μL of various concentrations of MEO and C10.

### 4.4. Agar Dilution Method 

The inhibitory activities of the MEO and C10 were determined by the agar dilution method [42]. Twenty-five mL of PDA medium was poured into Petri dishes (90 × 15 mm) at temperatures between 45–50 °C, and MEO and C10 dissolved in DMSO were diluted to obtain the following concentrations: 0.05, 0.1, 0.5, 1, 2.5, and 10 mg/mL. The negative control was prepared using DMSO, and the positive control (azoxystrobin) was spiked to concentrations of 0.1 and 0.2 mg/mL. A spore suspension (10^7^ spores/mL), which is collected from a 7-day-old fungal culture, was pipetted onto paper discs (6 mm). The fungal-inoculated paper discs were placed on the chemical-treated PDA medium in the center of the petri dishes. Each of the petri dishes was incubated at 25 ± 1 °C for 7 days, and mycelial growth was measured daily.

### 4.5. Mycelial Growth Assay

Assays for mycelial growth were conducted in 100 mL Erlenmeyer flasks containing 25 mL of the PDB liquid medium [25]. Each of the Erlenmeyer flasks was treated with 250 μL of MEO and C10 in the concentration range of 5–400 μg/mL, and *A. flavus* was inoculated with spore suspensions (10^7^ spores/mL). The mycelia of *A. flavus* were cultivated for 5 days in a shaking incubator at 25 ± 1 °C under 120 rpm. After incubation for 5 days, the mycelia of *A. flavus* were collected and filtered using Whatman No. 2 filter paper (185 mm in diameter). To measure the dry weight, the mycelia were then dried in a dry oven at 50 °C for 24 h to reach a complete dryness. In regard to evaluating the inhibitory activity of MEO and C10, this cultivation procedure, as described above, was applied to get *A. flavus* mycelia, and the mycelia of *A. flavus* was used to measure aflatoxin production and gene expression using qRT-PCR.

### 4.6. Aflatoxin Analysis Using Liquid Chromatography Triple Quadrupole Mass Spectrometry

The aflatoxins were extracted using the solvent ethyl acetate. The dryness of ethyl acetate extracts was performed using a rotary evaporator, and the dried extracts were re-dissolved in a mixture of methanol and water (1:1). Finally, they were filtered through a 0.20 μm microporous membrane for subsequent analysis using a liquid chromatography triple quadrupole mass spectrometer (LC-MS/MS, Agilent Technologies, Santa Clara, CA, USA). Two different mobile phases, water and methanol, were investigated. Each of the two solvents contained ammonium formate (5 mM) and formic acid (0.1%). The mobile phase was fixed to 0.3 mL/min for the flow rate, and the injection volume was set up to 5.0 µL. The temperature of the column oven was maintained at 40 °C. The analyses of AFB_1_ type were conducted using the HPLC system Agilent 1260 Infinity connected to an Agilent Jetstream electrospray ionization source (ESI), including a 6460 series Triple quadrupole (Agilent Technologies, Waldbronn, Germany). Then, 5.0 µL of the sample was injected into an Agilent Poroshell 120 SB-C18 column with a diameter (3.0 mm) and length (100 mm). For the detection of MS/MS, the ESI interface was operated in positive polarity, and other settings were 3.5 kV for the capillary voltage, 6 L/min for gas flow, 300 °C for source temperature, 500 V for nozzle voltage, 35 psi for nebulizer gas pressure, 11 L/min for sheath gas flow, and 250 °C for sheath gas temperature. A description of the LC-MS/MS analysis procedure is shown in Table S4. 

### 4.7. GC–MS Analysis and Quantification of Massoia Essential Oil

GC–MS (gas chromatography–mass spectrometry) analysis was undertaken on a Shimadzu Nexis GCMS 2030 spectrometer equipped using a split/splitless injector and a separation column (SH-Rtx-5MS, 30 m × 0.25 mm, 0.25 µm film) or a fused silica capillary column (FAMEWAX, 30 m × 0.25 mm, 0.25 µm film). Helium (99.999%) (BOC, North Ryde, NSW, Australia) was used as the carrier gas at a flow rate of 1.5 mL/min. An aliquot (1.0 μL) of MEO in *n*-hexane (40 µg mL^−1^) was injected in the split mode at a 30:1 ratio, with the injector temperature being 250 °C. The temperature program was set initially at 60 °C for 1 min, increased to 260 °C at a rate of 10 °C/min, and held for 3 min. The ion source and transfer line temperatures were 230 °C and 250 °C, respectively. The ionization method was electron impact (70 eV). Spectral results were obtained over a mass range of *m*/*z* 35–600. RIs were calculated by analyzing the *n*-alkane series (C8–C40) with SH-Rtx-5MS or FAMEWAX under the same GC operating conditions. For the identification of chemicals, the mass spectra were analyzed using the Shimadzu GCMS Postrun and compared with authentic samples, if available, NIST library (NIST17-1, NIST17-2, NIST17s) search, mass fragmentation patterns, and RIs published in the literature. 

### 4.8. Total RNA Isolation and qRT-PCR 

*A. flavus* mycelia in the PDB medium were closely harvested by filtering them using a cell strainer (SPL Life Sciences, Pocheon, Republic of Korea). The harvested *A. flavus* mycelia were sonicated for 5 min, left in a mortar, and ground into a fine powder using liquid nitrogen. Total RNA in the grounded mycelia was extracted with a Trizol reagent purchased from Invitrogen™ (Seoul, Republic of Korea). Quantitative measurement of extracted RNAs was performed by the determination of the absorbance at both 260 and 280 nm by a μDrop™ Plate supplied by Thermo Fisher Scientific (Waltham, MA, USA). RNA was qualitatively determined using an agarose gel electrophoresis (2%), including ethidium bromide. Complementary DNA (cDNA) was made using a Maxima First Strand cDNA Synthesis Kit (Thermo Fisher Scientific Inc.). Quantitative PCR was conducted at the KNU NGS Center (Daegu, Republic of Korea). A Luna Universal qPCR master mix obtained from New England BioLabs (Ipswich, MA, USA) with the prepared cDNA (1000 ng) was utilized for qRT-PCR analysis. Specific primers for the qRT-PCR analysis synthesized by Genotech (Daejeon, Republic of Korea) were used to determine the antiaflatoxigenic properties of MEO and C10. Twelves of primers were used, and they are *β-tubulin* and *erg28*, and aflatoxin-producing related genes such as *aflC*, *aflD*, *aflE*, *aflG*, *aflK*, *aflL*, *aflO*, *aflQ*, *aflR*, and *aflS* (Table S3). The operation process for the amplification was as follows: a denaturation step (95 °C for 30 s), an annealing step (60 °C for 20 s), and an elongation step (72 °C for 30 s), finally followed by 40 amplification rounds of a thermal cycling run with a post-cycling step (95 °C for 5 min). qRT-PCR analysis was undertaken three times for each sample. Measured differences in gene expression were re-calculated with the delta-delta Ct method. The results were evaluated and normalized with the *β-tubulin* gene expression, and gene expression comparisons were conducted. 

### 4.9. Statistical Analysis 

All experiments were performed three times. Results are expressed as mean ± standard deviation (SD). One-way analysis of variance (ANOVA) with post-hoc Tukey’s test was applied to analyze the results of inhibitory effects of natural products using the software package SPSS version 16.0, and significant differences between the control and chemical-treated samples were determined using one-way ANOVA (*p* < 0.05 level).

## Figures and Tables

**Table 1:** Identified compounds in massoia essential oil. MM: molar mass; RI: retention index; Ref: reference RI values from similar GC conditions. 5% represents the portion of the diphenyl functional group in the column.

No	Compound	MM	RI (5%)	RI (Wax)	Ref (5%)	Ref (Wax)	%
1	furfural	96.084	832	1450	835	1468	0.2
2	(*E*)-1,3-nonadiene	124.223	926	1257	924		0.8
3	5-methyl-2-furancarboxaldehyde	110.111	962	1557	963	1555	0.2
4	2-methoxyphenol	124.137	1086	1839	1090	1846	0.1
5	linalool	154.249	1099	1538	1098	1537	0.2
6	1,4-undecadiene	152.277	1126	1257			0.7
7	trans-3-nonen-2-one	140.223	1138	1523	1137	1500	0.1
8	1,3,5-undecatriene	150.261	1185	1402	1187		0.1
9	ylangene	204.351	1378	1480	1373	1485	0.1
10	copaene	204.351	1382	1488	1384	1488	0.2
11	1-ethenyl-1-methyl-2,4-bis(1-methylethenyl)-cyclohexane	204.351	1388	1585			0.1
12	methyl eugenol	178.228	1399	1995	1401	2020	0.3
13	β-bergamotene	204.351	1442	1581	1436	1586	0.1
14	γ-muurolene	204.351	1453	1682			0.2
15	alloaromadendrene	204.351	1472	1636	1467	1639	0.3
16	C10 massoia lactone	168.233	1485	2210	1483		45.2
17	δ-decalactone	170.249	1495	2168	1494	2160	1.2
18	β-bisabolene	204.351	1512	1721	1512		0.3
19	δ-Cadinene	204.351	1526	1751	1528		0.2
20	cis-calamenene	202.335	1530	1824	1532	1839	3.2
21	C12 massoia lactone	196.286	1697	2442			36.7
22	δ-dodecalactone	198.302	1709	2399	1711		1.9
23	benzyl benzoate	212.244	1776	2606	1770	2566	3.8
24	benzyl salicylate	228.243	1880	2762	1870	2737	0.3
25	C14 massoia lactone	224.339	1907	2673	1910		1.4
Total							98.9

## Data Availability

Data will be made available on request.

## Supplementary Materials

The following supporting information can be downloaded at https://www.mdpi.com/article/10.3390/toxins15090571/s1, Figure S1: Antifungal activities of massoia essential oil at the treated concentration of 50 and 500 mg/mL on *Aspergillus flavus* ATCC 22546 during 4 day incubation. Antifungal activities were measured with diameters (mm) of fungal growth zones in a potato dextrose agar (PDA) medium after treatments of massoia essential oil. Azoxystrobin at the concentration of 1 mg/mL was used as a positive control for this study. Diameters of fungal growth zones were measured every day. All experiments were performed in triplicates. Statistical analyses were performed using one-way ANOVA at the *p* < 0.05 level. (a) Schematic diagram of the antifungal measurement. (b) Picture of antifungal activities of massoia essential oil according to the incubation time (day). (c) Inhibition zones of fungal growth with statistical analysis. Table S1: Inhibition rate compared with positive control (%) of massoia essential oil (MEO) and C10 massoia lactone (C10) on *Aspergillus flavus* ATCC 22546 growth in disc diffusion assay. Table S2: Inhibition rate (%) of massoia essential oil (MEO) and C10 massoia lactone (C10) on *Aspergillus flavus* ATCC 22546 growth in agar dilution method. Table S3. List of primers used for qRT-PCR. Table S4: Liquid chromatography triple quadrupole mass spectrometer (LC-MS/MS) parameters for the analysis of aflatoxin B_1_, B_2_, G_1_, and G_2_ in multiple reactions monitoring mode.
