# Supplementary material for: Antifungal and Antiaflatoxigenic Activities of Massoia Essential Oil and C10 Massoia Lactone against Aflatoxin-Producing Aspergillus flavus
Source: Toxins (Basel). 2023 Sep 16;15(9):571. doi: 10.3390/toxins15090571 (PMC10537029; doi:10.3390/toxins15090571)
Supplement: Supplementary file 1 [file toxins-15-00571-s001.zip › toxins-2584803-supplementary.pdf]

toxins

Supplementary Data

Antifungal and Antiaflatoxigenic Activities of Massoia Essential Oil and C10 Massoia Lactone against Aflatoxin-Producing *Aspergillus flavus*

Yubin Lee, Soo Jean Park, Kyeongnam Kim, Tae-Oh Kim and Sung-Eun Lee

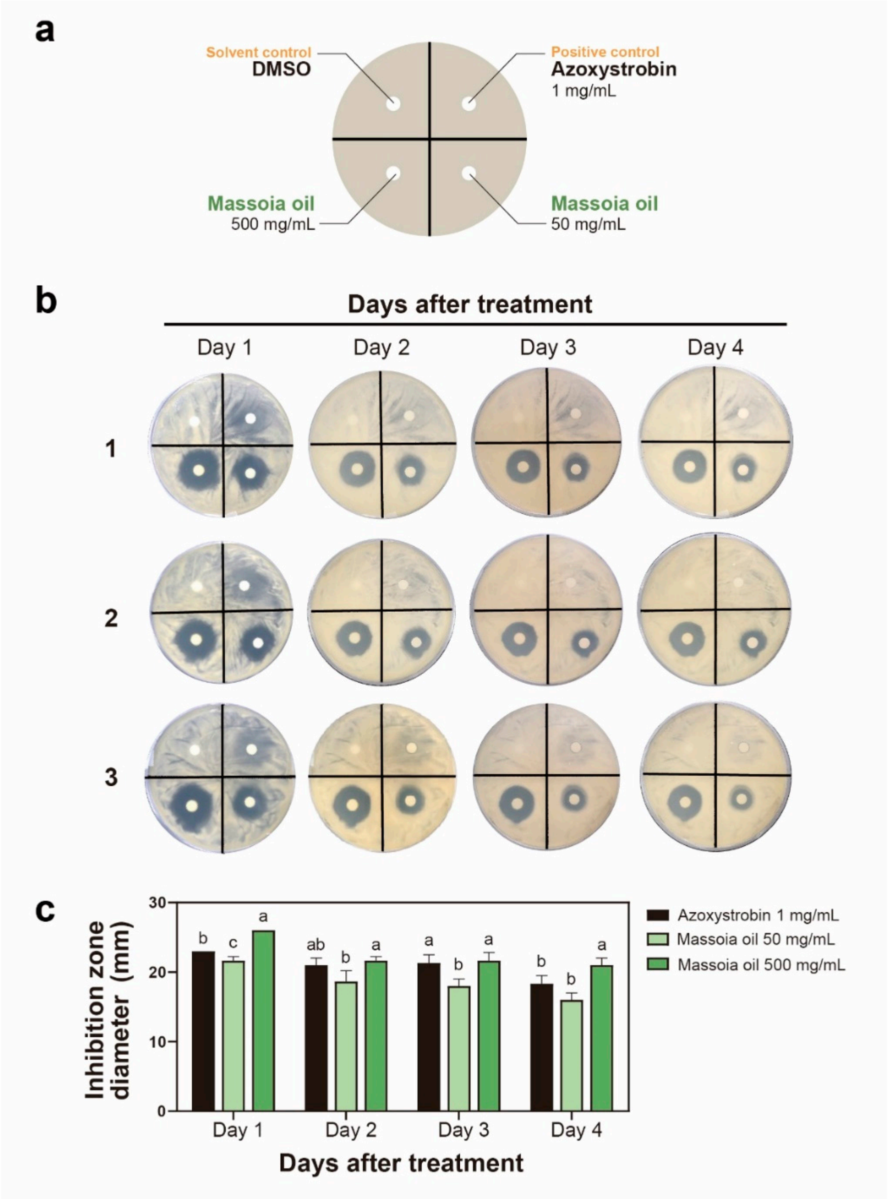

Figure S1. Antifungal activities of massoia essential oil at the treated concentration of 50 and 500 mg/mL on *Aspergillus flavus* ATCC 22546 during 4 day-incubation. Antifungal activities were measured with diameters (mm) of fungal growth zones in a potato dextrose agar (PDA) medium after treatments of massoia essential oil. Azoxystrobin at the concentration of 1 mg/mL was used as a positive control for this study. Diameters of fungal growth zones were measured every day. All experiments were done in triplicates. Statistical analyses were done using one-way ANOVA at the $p < 0.05$ level. (a), schematic diagram of the antifungal measurement. (b), picture of antifungal activities of massoia essential oil according to the incubation time (day). (c), inhibition zones of fungal growth with statistical analysis.

Table S1. Inhibition rate compared with positive control (%) of massoia essential oil (MEO) and C10 massoia lactone (C10) on *Aspergillus flavus* ATCC 22546 growth in disc diffusion assay.

Chemicals	Treated Concentration (mg/mL)	Inhibition Rate (%) ***
Negative control (DMSO *)	–	0
Positive control (Azoxystrobin **)	1	100
MEO	0.5	0
	2.5	45.0±0.0
	5	70.0±4.7
	10	75.0±5.3
	25	78.8±3.1
	50	92.5±1.8
C10	0.5	0
	2.5	41.7±4.2
	5	45.0±4.2
	10	67.6±2.8
	25	82.2±1.6
	50	91.2±2.8

* DMSO also called dimethylsulfoxide is employed to dissolve MEO and C10. ** Azoxystrobin was used as a positive control and it was used to determine the relative inhibition rates of MEO and C10. *** Inhibition rate compared with positive control (%) was calculated by comparing positive control with each diameter of inhibiting zone at 1 day after treatment. Inhibition rate compared with positive control (%) == $\frac{D}{P.C} \times 100(\%)$. D: Diameter of inhibition zone (mm) in the treated plate. P.C: Diameter of inhibition zone (mm) in the positive control plate.

Table S2. Inhibition rate (%) of massoia essential oil (MEO) and C10 massoia lactone (C10) on *Aspergillus flavus* ATCC 22546 growth in agar dilution method.

Chemicals	Treated Concentration (mg/mL)	Inhibition Rate (%) ***
Negative control (DMSO *)	–	0
Positive control (Azoxystrobin **)	0.1	27.5±0.7
	0.2	35.2±3.7
MEO	0.05	6.7±7.5
	0.1	17.4±4.4
	0.5	88.7±1.7
	1	100±0.0
	2.5	100±0.0
	10	100±0.0
C10	0.05	4.4±7.3
	0.1	17.4±13.5
	0.5	100±0.0
	1	100±0.0
	2.5	100±0.0
	10	100±0.0

* DMSO also called dimethylsulfoxide is employed to dissolve MEO and C10. ** Azoxystrobin was used as a positive control and it was used to compare the relative inhibition rates of MEO and C10. *** Inhibition rate (%) was calculated by comparing negative control with each mycelial diameter at 7 day after treatment. Inhibition rate (%) = $\frac{C-T}{C-d} \times 100(\%)$. C: Mycelial diameter (mm) in the control plate. T: Mycelial diameter (mm) in the treatment plate. d: Diameter (mm) of *A. flavus* mycelial discs.

Table S3. List of primers used for qRT-PCR.

Gene Symbol	Gene Function		Primer Sequences (5′-3′)
β-tubulin	Housekeeping gene	F	CTCCGTTCCTGAGTTGACCC
		R	GAGCAGGTGAGGTAACGACC
aflC	Polyketide synthase	F	ACTGGCAACTGCAAACCCTA
		R	CCAGCCGTTTGATGAACACC
aflD	Reductase	F	CCAACATGCACGACTATGCG
		R	GCCGTGAGCCATTTGTTCTC
aflE	NOR reductase	F	CGTCTCTCAGTCAAGGCCAG
		R	TCGCATCACTTCCTCCACAC
aflG	P450 monooxygenase	F	GCATCTTCCACCCTTCCACA
		R	GAAAAGGCCAACAGTCGTCG
aflK	VERB synthase	F	ATGCAGGGAAAGACCTTGGG
		R	AACTATCGTCGCCAACGTGA
aflL	Desaturase	F	GCAACAGTTTGTGGCCGATT
		R	ATGAACTTGTCGGCGTGAGT
aflO	*O*-methyltransferase B	F	AATTCCCCGCTCCTGACAAG
		R	CGACCAGGAAGGTTGGGAAA
aflQ	Oxidoreductase	F	GATAACCCGGACGACCTTCG
		R	CTCATCTTTTCCATGCGGCG
aflR	transcription regulator	F	TGCAGTCAATGGAACACGGA
		R	TGGGGGTCCCTACTTCCAAA
aflS	transcription regulator	F	GTGGAGGATACGCTCACTCG
		R	GTGATGGAAATTGCGGAGCG
erg28	14-*α*-demethylase	F	TTGCCTCCTTTTGAGGGCTT
		R	CACAGGGGTCGTGATGTTGT

Table S4. Liquid chromatography triple quadrupole mass spectrometer (LC-MS/MS) parameters for the analysis of aflatoxin B_1, B_2, G_1, and G_2 in multiple reactions monitoring mode.

Aflatoxin	Molecular Formula	Molecular Weight (g/mol)	Retention Time (min)	Precursor Ion $[M+H]^+$ (m/z)	Production (m/z)	Dwell Time (ms)	Fragmentor Voltage (V)	Collison Energy (V)
B_1	$C_{17}H_{12}O_6$	312	8.80	313.1	285 *	200	100	20
					241			40
B_2	$C_{17}H_{14}O_6$	314	8.61	315.1	287.1 *	200	100	35
					259.1			35
G_1	$C_{17}H_{12}O_7$	328	8.32	329.1	243 *	200	110	25
					200			45
G_2	$C_{17}H_{14}O_7$	330	8.13	331.1	313 *	200	110	25
					245.1			30

* Quantification product ions.
